# Nutrient Loading Fosters Seagrass Productivity Under Ocean Acidification

**DOI:** 10.1038/s41598-017-14075-8

**Published:** 2017-10-23

**Authors:** Chiara Ravaglioli, Chiara Lauritano, Maria Cristina Buia, Elena Balestri, Antonella Capocchi, Debora Fontanini, Giuseppina Pardi, Laura Tamburello, Gabriele Procaccini, Fabio Bulleri

**Affiliations:** 1 Dipartimento di Biologia, Università di Pisa, CoNISMa, Via Derna, 1, 56126 Pisa Italy; 2 Stazione Zoologica Anton Dohrn, Villa Comunale, 80121 Napoli, Italy; 3 Present Address: CoNISMa, Piazzale Flaminio, 9, 00196 Roma, Italy

## Abstract

The effects of climate change are likely to be dependent on local settings. Nonetheless, the compounded effects of global and regional stressors remain poorly understood. Here, we used CO_2_ vents to assess how the effects of ocean acidification on the seagrass, *Posidonia oceanica*, and the associated epiphytic community can be modified by enhanced nutrient loading. *P. oceanica* at ambient and low pH sites was exposed to three nutrient levels for 16 months. The response of *P. oceanica* to experimental conditions was assessed by combining analyses of gene expression, plant growth, photosynthetic pigments and epiphyte loading. At low pH, nutrient addition fostered plant growth and the synthesis of photosynthetic pigments. Overexpression of nitrogen transporter genes following nutrient additions at low pH suggests enhanced nutrient uptake by the plant. In addition, enhanced nutrient levels reduced the expression of selected antioxidant genes in plants exposed to low pH and increased epiphyte cover at both ambient and low pH. Our results show that the effects of ocean acidification on *P. oceanica* depend upon local nutrient concentration. More generally, our findings suggest that taking into account local environmental settings will be crucial to advance our understanding of the effects of global stressors on marine systems.

## Introduction

Interactive and cumulative effects of global climate changes resulting from enhanced CO_2_ emissions, such as seawater warming and ocean acidification (OA), have been extensively investigated in the last decade^1,2^. In contrast, despite the recognition that human stressors on natural systems build-up from global to local scales, less attention has been devoted to assess how the effects of global changes on marine systems can be modified by a regional or local stressor^3,4^.

Anthropogenic ocean acidification is among the major climate-related stressors in marine coastal ecosystems (IPCC 2014). Increased partial pressure of CO_2_ (*p*CO_2_) in seawater can have both positive and negative impacts on marine primary producers^5,6^. Negative effects have been widely recorded for calcifying macroalgae^7,8^. Seagrasses are generally carbon-limited under current *p*CO_2_, and may benefit from an increment in CO_2_ and HCO_3_^6^, although their response can differ among species as a result of variation in carbon concentration mechanisms^9^. The increase in DIC and CO_2_ associated to OA can enhance nitrogen demand for plants^10,11^. Previous studies on terrestrial plants have shown that the initial stimulation of photosynthesis and growth at elevated CO_2_ levels were subsequently down-regulated as nitrogen content decreased^10,12^. Therefore, the net effect of OA on seagrasses may depend upon nitrogen availability.

Enhanced nutrient loading in coastal environments could be expected to generate^13^ or amplify^14^ positive effects of OA on the productivity of seagrasses. Mesocosm studies have found little evidence of positive effects of OA on seagrasses to be sustained by nutrient enhancement^9,15–17^. The duration of these studies (24 days to 6 weeks) could be too short for enhanced *p*CO_2_ to induce nutrient limitation, although responses are likely species-specific, depending on the plant metabolism and growth dynamics. In addition, the effects of nutrients have been generally assessed by comparing the performance of seagrasses between nutrient ambient *versus* nutrient enhanced conditions, providing no information on how they can vary according to their concentration. Whether enhanced nutrient availability may alleviate N limitation at low pH, excessive loading could negatively affect seagrasses through the reduction of carbon reserves and internal imbalance of other essential nutrients (e.g. C and P)^18,19^.

OA can also affect seagrasses indirectly, through the modification of biotic interactions^3,16^. At ambient pH, moderate or high nutrient concentrations have been widely shown to cause seagrass decline by promoting excessive epiphyte proliferation^20,21^. At pH levels predicted for the end of the century, the marked decline in the calcifying component of the epiphytic assemblage^22–24^ may expose shallow-water plants to excessive light and UV radiation stress^25,26^. Under these circumstances, nutrient-induced proliferation of epiphytes might be beneficial for the plants.

Plants react to stressful conditions, such as those induced by intense light, activating a series of response mechanisms, in which many of the genes involved are highly conserved across species. Stress-responsive genes include antioxidant and macromolecule damage sensors and genes coding for proteins that adjust cellular physiology and metabolism in response to specific stressors. Recently, Lauritano, *et al*.^27^ compared the expression of genes involved in the response to stress between plants collected at low natural pH, near volcanic vents, and plants collected at normal pH conditions. These authors found increased expression of some antioxidant and stress-related genes in epiphyte-free leaves of *P. oceanica* close to vents, suggesting overall negative effects of increased CO_2_ and low pH on plant physiology, possibly triggered by extensive leaf epiphyte loss^22^. In this light, long-term studies encompassing seasonal variations in plant metabolism^28^ and epiphytic assemblage structure^29^, as well as multiple levels of nutrient loading, appear necessary to improve our understanding of seagrass response to OA.

In this study, we used CO_2_ vents along the coast of Ischia Island (Italy) to assess how the effects of OA on the seagrass, *P. oceanica*, and the associated epiphytic community can be modified by different nutrient concentrations. Submarine vents are characterized by the emissions into seawater of gases, predominantly CO_2_, and they can be used to study the effects of naturally acidified seawater on biological communities as there are no confounding gradients of temperature, salinity, hydrodynamic conditions and toxic hydrogen sulphide^14,30,31^. In particular, previous studies carried out at Ischia Island vents have extensively demonstrated that areas exposed to CO_2_ bubbling do not differ from control areas in terms of temperature, hydrodynamic conditions, salinity, light and total alkalinity, by virtue of the fact that they are just 10 s of m apart^14,30,32,33^. As a consequence of temporal fluctuations in pH and the presence of other components deriving from the volcanic activity, vents are not perfect predictors of OA effects on marine systems^14,27,34^. On the other hand, they allow the integration of species interactions and seasonal cycles, unlikely to be achieved under laboratory settings.

We exposed the slow-growing seagrass, *P. oceanica*, at either current sea pH (hereafter referred to as ambient pH) or expected future pH (hereafter referred to as low pH), to different nutrient concentrations (control, moderate and high enhancements) for 16 months. We assessed the response of *P. oceanica* to different combination of pH and nutrient loading at the individual and community level, combining analyses of gene expression, plant growth, photosynthetic pigment concentrations and epiphytic assemblage structure. The analysis of gene expression provides important insight for understanding how organisms react to environmental stimuli. We selected target genes involved in response to stress (e.g. enzymes involved in the metabolism of the scavenger molecule glutathione, detoxification of free radicals and general antioxidant) and in the transport of nitrogen thorough plant tissues.

The effects of nutrient enrichment on the seagrass were expected to vary between ambient and low pH sites. Specifically, we hypothesized that under nutrient concentrations unlikely to cause N- or P-limitation (such as those found at our study site; see Materials and Methods section), both moderate and high nutrient enrichment at ambient pH would negatively affect seagrass productivity, either directly (e.g. carbon reserves depletion)^18^ or indirectly (e.g. via epiphyte overgrowth)^20^. At low pH, potential stimulation of plant photosynthesis and growth can be hindered by either nutrient limitation^10,11^ or excessive light stress following leaf epiphyte loss^22,25,26^. In this case, a moderate increase in nutrient availability may promote seagrass productivity directly, likely by alleviating N-limitation, and, indirectly, by reducing light/UV stress through the restoration of the epiphytic community. A further increase of nutrient enrichment could be instead expected to have negative effects on plants also at low pH, as a consequence of the excessive proliferation of leaf epiphytes. At the genetic level, we expected that plant exposure to high nutrient loading and low pH could either result in increased expression of stress-responsive genes, due to the cumulative effects of the two stressing conditions, or to the lowering of the antioxidant response, due to a positive effect of N addition at high C concentration. Along this line, the expression of nitrogen transporters was expected to increase as well at low pH, if plants were able to take advantage of enhanced nutrient loading.

## Results

### Expression levels of the genes of interest (GOI)

**Figure 1:**
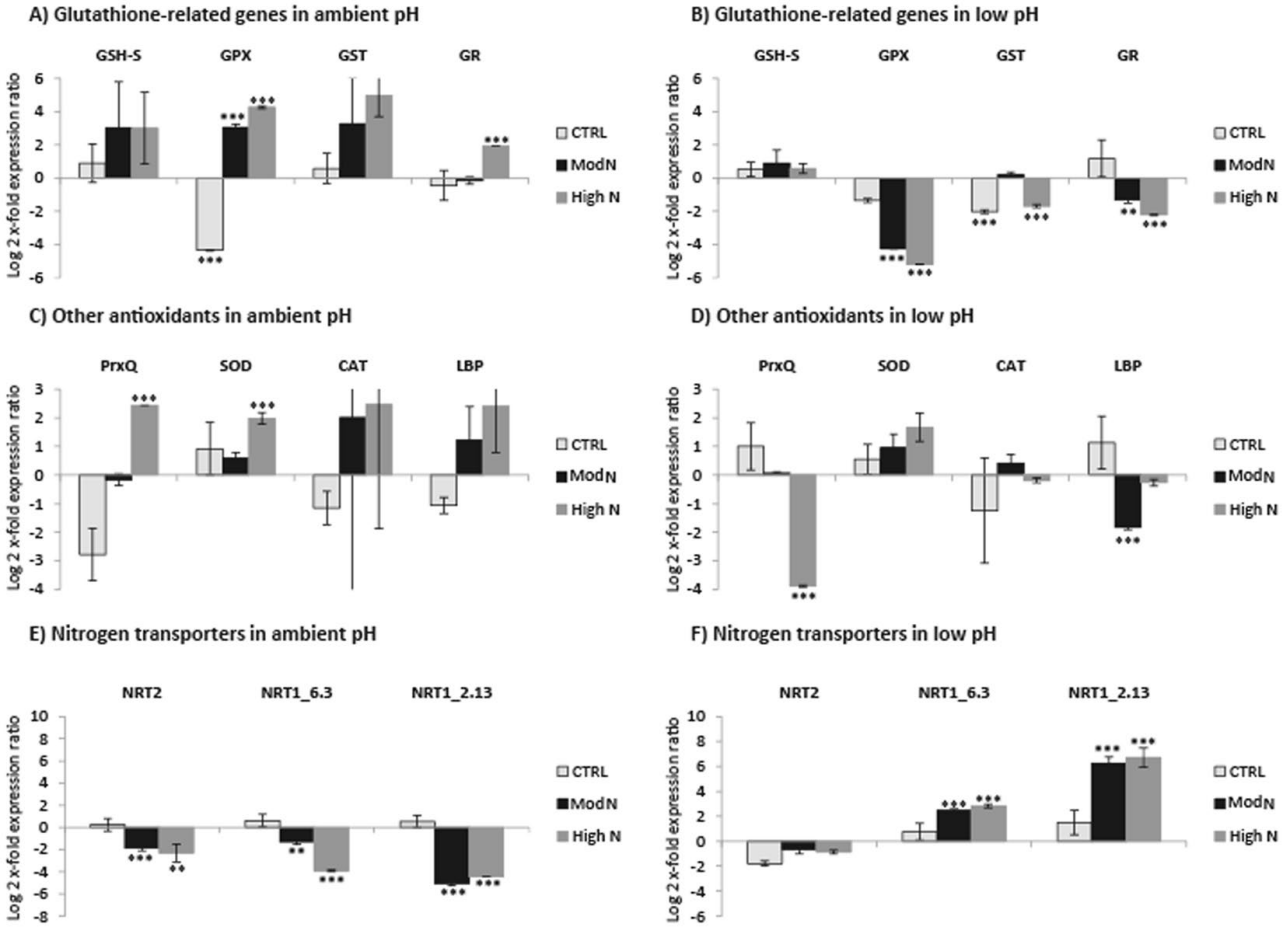
Expression levels of the selected genes of interest at ambient pH or in the vicinity of the CO_2_ vents under three nutrient regimes (CTRL = control, High N = high nutrient enrichment; Mod N = moderate nutrient enrichment), using as control condition plants collected at time 0 (represented in figure by the x-axis). Genes are grouped for gene categories in glutathione-related genes at the ambient (**A**) and low pH sites (**B**), other antioxidant genes at the ambient (**C**) and low pH sites (**D**), and nitrogen transporter genes at the ambient (**E**) and low pH sites (**F**).

In order to assess the effects of experimental conditions on the expression level of selected GOI (grouped in glutathione-related genes, other antioxidants and nitrogen transporters), gene expression in *P. oceanica* plants collected after 16 months (July 2015) since the start of the experiment was compared with the expression of plants collected in the same plot at time 0 (May 2014, before adding nutrients; represented by the x-axis in Fig. 1). Glutathione-related genes were up-regulated at ambient pH (Fig. 1A; *p* < 0.001 for GPX at both moderate and high nutrient levels and for GR at high nutrient levels) and were down-regulated at low pH (Fig. 1B; *p* < 0.001 for GPX in both moderate and high nutrient enrichment and for GST and GR at high nutrient levels, *p* < 0.01 for GR at moderate nutrient levels). In plots not exposed to nutrient addition (CTRL), there were no significant changes, except for GPX that was down-regulated at ambient pH condition (Fig. 1A; *p* < 0.001) and GST down-regulated at low pH (Fig. 1B; *p* < 0.001). The expression of other antioxidant genes, such as PrxQ and SOD, involved in free radical detoxification, increased under high nutrient enrichment at ambient pH (Fig. 1C; p < 0.001 for both), while CAT and LBP did not show significant changes. At low pH (Fig. 1D), PrxQ was significantly down-regulated at high nutrient levels (*p* < 0.001) and LBP was down-regulated under moderate nutrient enrichment (*p* < 0.01). The expression of other antioxidant genes did not change significantly. In plots without nutrient addition (CTRL), there were no significant changes in antioxidant genes expression. The expression of the three nitrogen transporter genes decreased under moderate and high nutrient enrichment at ambient pH (Fig. 1E; *p* < 0.001 for NRT2 under moderate nutrient enrichment, NRT1_6.3 under high nutrient enrichment and NRT1_2.13 under both moderate and high nutrient enrichment; *p* < 0.01 for NRT2 under high nutrient enrichment and NRT1_6.3 under moderate nutrient enrichment). In contrast, at low pH, NRT2 expression levels did not change significantly, while NRT1_6.3 and NRT1_2.13 were both up-regulated under moderate and high nutrient enrichment (Fig. 1F; *p* < 0.001 for all). In plots not exposed to nutrient addition (CTRL), there were no significant changes in the expression of nitrogen transporter genes.

### Physiological response of *P. oceanica* to experimental treatments

**Figure 2:**
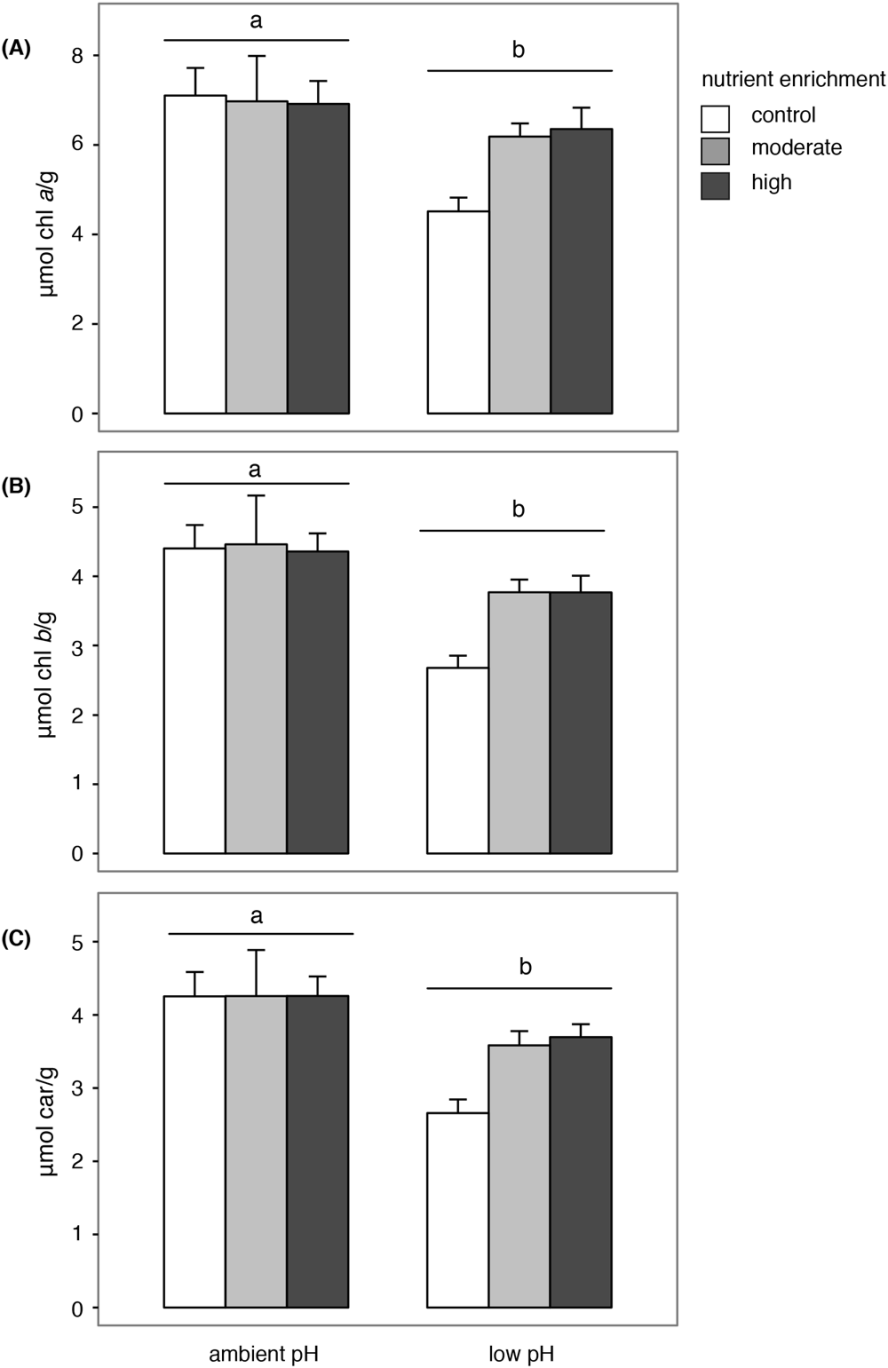
Mean content (μmol/g, +SE, n = 12) of (**A**) chlorophyll *a*, (**B**) chlorophyll *b* and (**C**) carotenoids at different combinations of pH (ambient and low pH) and nutrient enrichment (control, moderate, high). Different lower case letters indicate significant differences between pH treatments.

The concentration of pigments (µmol g^−1^ chlorophyll *a*, *b*, carotenoids) in *P. oceanica* leaves varied according to pH conditions (Table 1). The content of chlorophyll *a*, *b* and carotenoids was higher at ambient than low pH. Although the effects of nutrient addition were not statistically significant, at low pH, there was a trend for pigment content to be higher under elevated than ambient nutrient levels (Fig. 2A,B,C). In particular, a moderate supply of nutrients caused an increment of ~27% of chlorophyll *a*, ~29% of chlorophyll *b* and ~26% of carotenoids. Likewise, a high supply of nutrients resulted in ~29% of chlorophyll *a* and *b* and ~28% of carotenoids.

**Table 1:** ANOVAs on the effects of pH (ambient and low pH) and nutrient enrichment (control, moderate and high) on the leaf content of chlorophyll *a*, chlorophyll *b*, carotenoids, the leaf growth and specific leaf growth. Analyses of the percentage cover of encrusting coralline algae, non-encrusting algae and total epiphytic assemblages on *P. oceanica* leaves also include the factor plot (3 levels, random and nested within the interaction pH × Nutrients). *P < 0.05; **P < 0.01; ***P < 0.001. SNK tests are included for significant factors or “pH × Nu” interaction.

	df	MS	*F*	
**Chlorophyll** ***a***				
pH	1	7.746	7.440**	
Nutrient (Nu)	2	1.279	1.228	
pH × Nu	2	1.848	1.775	
Residual	12	1.041		
**Chlorophyll** ***b*** ^**I**^				
pH	1	0.192	12.096**	
Nutrient (Nu)	2	0.032	2.050	
pH × Nu	2	0.035	2.221	
Residual	12	0.016		
**Carotenoids**				
pH	1	4.007	11.788**	
Nutrient (Nu)	2	0.491	1.443	
pH × Nu	2	0.483	1.421	
Residual	12	0.34		
**Leaf growth**				SNK tests
pH	1	0.841	1.247	Control nutrient: ambient pH > low pH
Nutrient (Nu)	2	0.479	0.710	Moderate nutrient: ambient pH = low pH
pH × Nu	2	2.801	4.156*	High nutrient: ambient pH = low pH
Residual	12	0.674		
**Specific leaf growth** ^**II**^				SNK tests
pH	1	0.265	51.776***	^d^Ambient pH: control = moderate = high
Nutrient (Nu)	1	0.032	6.232**	Low pH: control = moderate < high
pH × Nu	2	0.019	^d^3.628*	
Residual	12	0.005		
**Encrusting coralline algae**				SNK tests
pH	1	48014	230.602***	^**e**^Ambient pH: control < moderate = high
Nutrient (Nu)	2	1584	7.606**	Low pH: control = moderate = high
pH × Nu	2	1436	^**e**^6.896**	
Plot (pH × Nu)	12	208	2.877**	
Residual	36	72		
**Total non-calcified algae** ^**II**^				
pH	1	35.876	^**f**^5.561*	^**f**^pH: ambient > low
Nutrient (Nu)	2	36.408	^**g**^5.643**	^**g**^Nutrient: control < moderate = high
pH × Nu	2	17.907	2.775	
Plot (pH × Nu)	12	6.452	3.032**	
Residual	36	2.128		
**Total epiphytic assemblage** ^**I**^				
pH	1	1.473	^**h**^38.499***	^**h**^pH: ambient > low
Nutrient (Nu)	2	0.450	^**i**^11.774**	^**i**^Nutrient: control < moderate = high
Ph × Nu	2	0.048	1.257	
Plot (pH × Nu)	12	0.038	1.570	
Residual	36	0.024		

^I^Data log (x + 1) transformed to satisfy parametric test assumptions.

^II^Data sqrt (x + 1) transformed to satisfy parametric test assumptions.

### Plant growth and epiphyte assemblage structure

**Figure 3:**
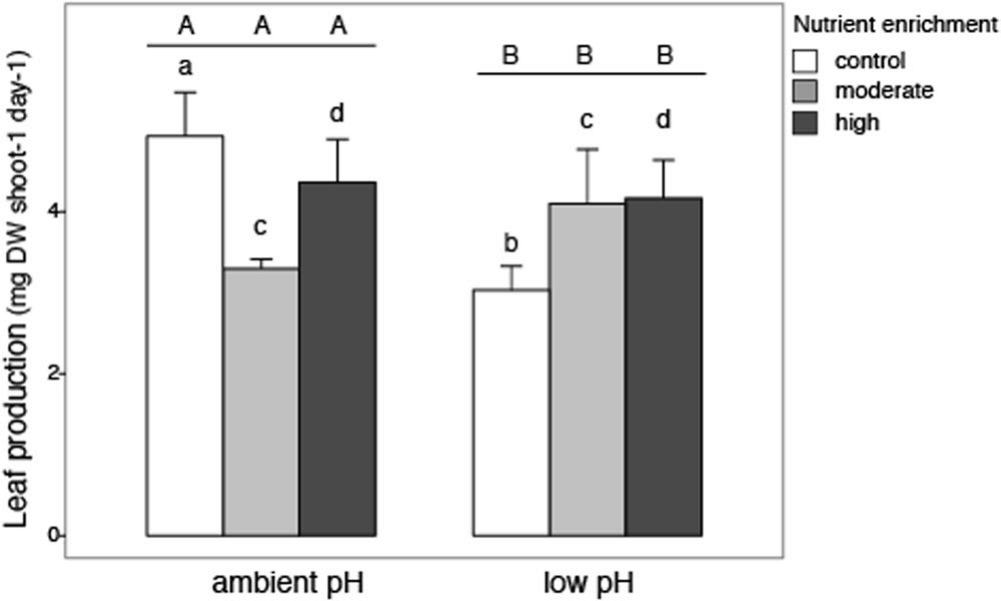
Leaf growth of *P. oceanica* (mg DW/ shoot day, mean, +SE, n = 9) for different combinations of pH (ambient and low pH) and nutrient enrichment (control, moderate, high). Letters above columns indicate the outcome of SNK tests; different letters indicate significant differences. Lower case letters show the comparison between pH treatments within each nutrient condition, while capital letters show the comparison among nutrient treatments within each pH condition.

There was a significant interaction between pH and nutrient enrichment on *P. oceanica* leaf growth (Table 1). Under control nutrient condition, leaf growth rate was higher at ambient than at low pH conditions while, under elevated nutrient enrichment, there were no differences in leaf growth between pH values (Fig. 3). At low pH, there was an increment (albeit not significant) of ~26% and ~27% of plant leaf growth under moderate and high nutrient supply, respectively. In addition, at low pH, the specific growth rate of *P. oceanica* was higher under high than moderate or control nutrient levels, while no differences were found among nutrient levels at ambient pH (Table 1; Supplementary Fig. S1).

**Figure 4:**
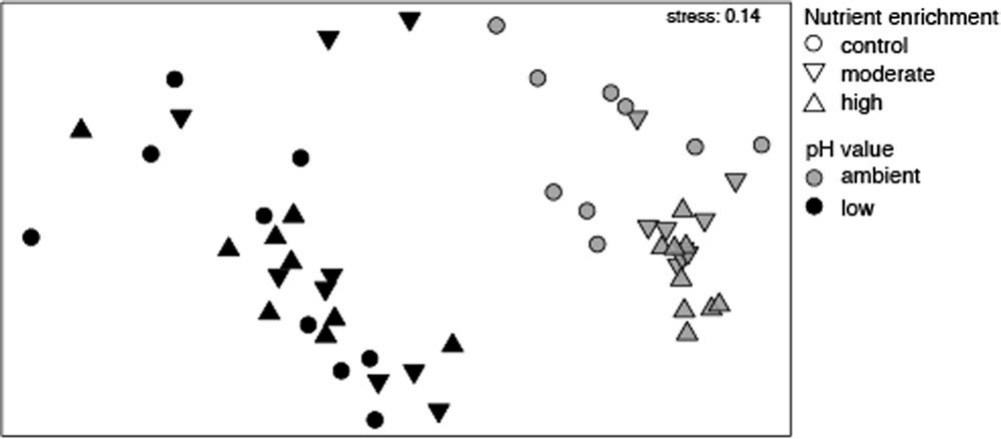
MDS ordination on untransformed data obtained from Bray-Curtis dissimilarities showing differences in leaf epiphytic assemblages between low pH and ambient pH (respectively *black* and *grey* symbols) and among nutrient enrichment levels (*circle* control nutrient, *triangle down* = moderate nutrient enrichment and *triangle up* = high nutrient enrichment**)**.

The structure of the epiphytic community on *P. oceanica* leaves was influenced by both pH and nutrient levels, but there were no effects of their interaction (PERMANOVA in Table 2a). The epiphyte assemblage differed between pH values and between elevated and control nutrient treatments (pairwise test for pairs of levels of factor nutrient) (Fig. 4). In addition, there were no significant differences in data dispersion for both pH and nutrient conditions, indicating that different experimental treatments did not cause changes in the spatial heterogeneity of epiphytic assemblage (PERMDISP in Table 2b).

**Table 2:** (a) PERMANOVA and (b) PERMDISP on the effects of pH (ambient and low pH), nutrient enrichment (control, moderate, high) and plot (3 levels) on the epiphyte assemblage on *P. oceanica* leaves. Pairwise tests for pairs of levels are reported for the factor Nutrient enrichment. *P < 0.05, **P < 0.01, ***P < 0.001.

(a) PERMANOVA			
Source of variation	df	MS	Pseudo-*F*
pH	1	40086	34.437***
Nutrient (Nu)	2	3094.8	2.659**
pH × Nu	2	1728.2	1.485
Plot (pH × Nu)	12	1164.1	1.781**
Residual	36	653.78	
Total	53		
***Pairwise test for pairs of levels of factor nutrient***	***t***	***P***	
High vs Control	2.028	0.008	
High vs Moderate	1.039	0.401	
Moderate vs Control	1.557	0.067	
**(b) PERMDISP: deviations from centroid**			
**Source of variation**	**Pseudo-** ***F***	***P***	
pH	2.977	0.126	
Nutrient	0.123	0.908

**Figure 5:**
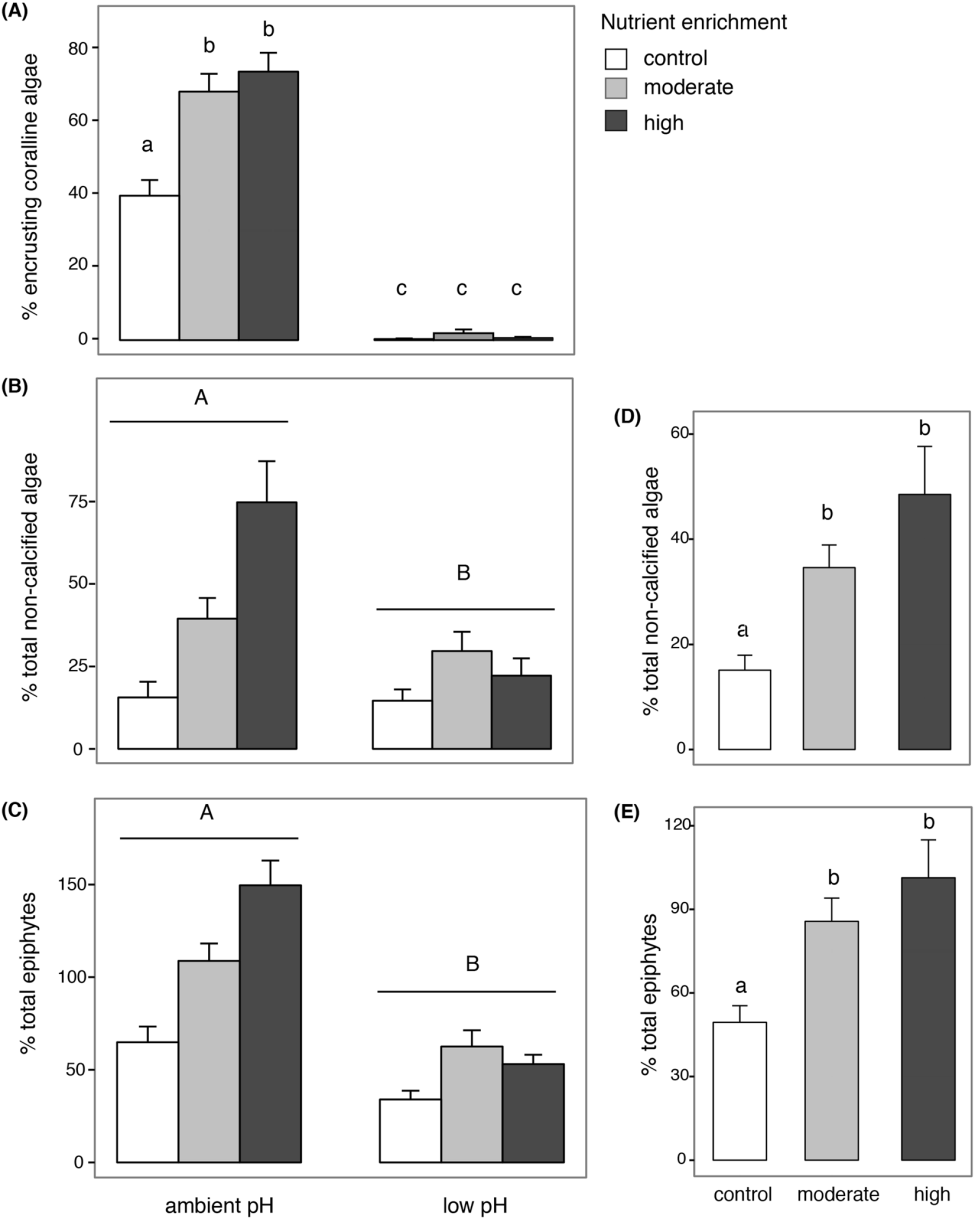
Mean percentage cover (+SE, n = 18) of (**A**) encrusting coralline algae, (**B**) non-calcified algae and (**C**) total of epiphytes for different combinations of pH (ambient and low pH) and nutrient enrichment (control, moderate, high). Lower case letters in (**A**) illustrate the outcome of SNK comparing nutrient treatments within each pH level, whilst capital letters in (**B**) and (**C**) indicate the differences between pH treatments. Percentage cover of (**D**) non-calcified algae and (**E**) total of epiphytes under different nutrient enrichment (control, moderate, high). Data are means + SE (n = 36 as pH conditions were pooled). Lower case letters illustrate the outcome of SNK comparing nutrient treatments. Different letters indicate significant differences.

There was a significant interaction between pH and nutrient enrichment on the cover of encrusting coralline algae: both moderate and high nutrient enrichment increased the cover of this group at ambient pH, while they had no effect at low pH (Table 1, Fig. 5A).

Total cover and cover of non-calcified algae (filamentous algae, foliose algae and the brown crust *Myrionema*) were greater at ambient than low pH (Table 1, Fig. 5B,C) and increased under both moderate and high nutrient enrichment (Table 1, Fig. 5D,E).

## Discussion

Long-term nutrient enrichment modulated the effects of ocean acidification on *P. oceanica*. At low pH, nutrient addition enhanced plant growth and photosynthetic pigment content. In contrast, at ambient pH, elevating nutrient levels increased the expression of selected antioxidant genes, indicating a response of plants to stressful conditions.

Seagrasses can be expected to benefit from OA since their productivity is likely carbon-limited under current CO_2_ concentration^6^. Short-term laboratory studies have shown an increase in seagrass productivity and biomass, as well as a decrease in chlorophylls and leaf nitrogen, at low pH^35,36^. In contrast, long-term experiments have found no differences in the growth rate of seagrasses at elevated CO_2_ concentration, possibly due to nutrient limitation^11,13,37^. Evidence from terrestrial studies suggests that the initial increase of plant productivity triggered by high CO_2_ levels can be reverted following photosynthesis acclimation, resulting in reduced growth and biomass of non-fertilized plants over longer periods of time^10^.

CO_2_ vents can be used to evaluate *in situ* the performance of plants adapted to long-term elevated CO_2_. Previous evidence from tropical and temperate volcanic vents suggests that seagrasses have successfully adapted to live under permanent low pH condition, resulting in higher shoot density and biomass compared to current pH levels^14,38,39^. Nonetheless, evidence of enhanced photosynthesis and growth near vents remain somewhat elusive^14,40,41^. In our study, nutrient supply may have released *P. oceanica* from nitrogen limitation, ultimately fostering leaf growth. Although we did not measure N leaf content, over-expression of nitrogen transporter genes (i.e. NRT1_6.3 and NRT1_2.13, in particular), caused by nutrient additions at low pH, suggests enhanced N intake by plants, likely in attempt to balance the C increase. At ambient pH, nitrogen transporter gene expression was down-regulated irrespective of nutrient levels, suggesting no need of increasing nitrogen uptake under current C concentration.

Pigment content in *P. oceanica* was smaller at low than ambient pH. This may be an acclimation strategy consisting in the degradation of excess light-harvesting pigments under nutrient limitation^42,43^. Supply of nutrients was somewhat effective in enhancing pigment concentration in *P. oceanica* leaves at low pH. Similar positive effects of nutrient addition on photosynthetic pigments have been reported for nutrient limited seagrasses in the tropics^44^ and suggest that chlorophylls and carotenoid synthesis in *P. oceanica* may be dependent on nutrient availability. As proposed by Agawin, *et al*.^44^ for tropical seagrasses, enhanced carbon assimilation by *P. oceanica* following nutrient deficiency alleviation may, at least in part, result from enhanced synthesis of photosynthetic pigments.

The positive effect of nutrient addition on the performance of marine plants at low pH is in contrast with previous findings from other temperate coastal regions. Evidence from kelp-dominated systems indicates that compounded effects of OA and nutrient enrichment can promote the proliferation of opportunistic filamentous algae at the expense of encrusting forms and, indirectly, of kelp^3^. Our results suggest that the effects of global climate change may depend on local environmental settings and species life-traits, thus complicating management strategies.

Ocean acidification and nutrient enrichment altered the structure of the epiphytic assemblage on *P. oceanica* leaves. Coralline algae are particularly susceptible to low pH, due to their high magnesium skeleton^8,14,22,23^. We documented a decline in the cover of encrusting coralline algae from ~40% at ambient pH to nearly 1% at low pH. Nutrient enrichment increased the abundance of encrusting coralline algae at ambient pH, but, in accordance with previous long-term studies^23^, they did not at low pH.

Non-calcified algal cover was smaller at low than ambient pH, in line with the findings of Martin, *et al*.^22^. Other studies have documented positive effects of OA on filamentous algal epiphytes^3,23^, inferring that these algal forms would be able to increase their photosynthetic rates under enhanced carbon concentration^45^. However, primary producer response to increased CO_2_ seems to be species-specific and there is limited information on the carbon acquisition pathway of many species^6^. The decrease of non-calcified epiphytes on *P. oceanica* leaves at low pH could be an indirect consequence of the loss of encrusting coralline algae that, acting as pioneers in the colonization of seagrass leaves^46^, may enhance their suitability as habitat for other epiphytic groups.

Nutrient-induced proliferation of leaf epiphytes at ambient pH has been recorded for a variety of seagrasses^47–49^, including *P. oceanica* ^21^. In our study, both moderate and high nutrient enrichment were effective in promoting the development of non-calcified epiphytic algae also at low pH, ultimately enhancing total epiphyte cover to values matching those found at ambient pH and nutrient concentration. Such a response is in contrast with the results of Campbell and Fourqurean^23^, who found no effect of long-term nutrient supply on the epiphyte assemblage supported by plants of the tropical seagrass, *Thalassia testudinum*, maintained at low pH. This contrast could be due to variations in life-traits (e.g. leaf-growth rates) between temperate and tropical seagrasses. Alternatively, it could be only apparent, as it may be the result of the different procedures used to enhance nutrient levels: in the case of Campbell and Fourqurean^23^, nutrient pellets were left loose on the bottom of plots, while, in this study, they were contained in mesh bags suspended within or slightly above the seagrass canopy (see methods). The release of nutrients directly into the water column may have had elicited a stronger response by the epiphytes growing on the leaves.

Overgrowth by epiphytes under eutrophic conditions is widely acknowledged among the major causes of seagrass decline^20^. However, the sign and strength of species interactions is not invariant, but likely to vary according to environmental conditions^50^ and to the relative abundance of interacting species^51^. Previous laboratory studies have shown that seagrasses may rely primarily on shading by a thick layer of leaf epiphytes to dampen the negative effects of high light intensity and UV radiation^25^. Thus, both the uncontrolled proliferation and the total loss of epiphytes are likely detrimental to seagrasses: in the first case, the plant would be light limited, in the second, it would be exposed to photo-inhibition due to excessive light intensity and damage from UV-B rays, especially in shallow water populations^25,26^. Hence, at low pH, the increase in the cover of leaf epiphytes generated by nutrient enrichment, although moderate, may have indirectly contributed to enhance leaf growth of *P. oceanica* by reducing light stress^52^. Indeed, although shallow *P. oceanica* plants are adapted to live in high-light conditions, they activate specific mechanisms of defence from excessive light, that are modulated during the daily cycle^53,54^. However, further manipulative experiments are needed to assess the role of epiphytes as light screen on seagrass leaves.

At low pH, we observed a down-regulation of the antioxidant LBP under moderate nutrient enrichment condition and of the glutathione-related enzymes GPX and GR under both moderate and high nutrient enrichment (Fig. 1). Likewise, there was a reduction in GST and PrxQ expression under high nutrient enrichment (Fig. 1). These patterns indicate that enhanced nutrient supply can cause a reduction in the stress response machinery in plants exposed to OA, even considering that plants close to the vents may be locally adapted to low pH.

At ambient pH, we found no effects of nutrient enrichment on *P. oceanica* leaf growth rate and photosynthetic pigment content. Seagrasses exposed to chronic nutrient enrichment often show reduced biomass and growth rate and enhanced epiphyte loading and shoot mortality^20^. However, these variables do not always respond consistently to nutrient enrichment and, in some cases, seagrasses can develop mechanisms to regulate nitrogen and phosphorus metabolism under elevated nutrient loading^55^ or accumulate excess nutrients in the rhizomes when plants are not nutrient-limited^56^. In addition, epiphytes can decrease nutrient uptake by seagrass leaves^57,58^ either by creating a physical barrier between the water column and active uptake sites on leaf surface or by a more rapid use of available nutrients^59,60^. In our study, the proliferation of epiphytes on seagrass leaves, generated by elevated nutrient enrichment at ambient pH, did not translate into negative effects on the leaf growth rate or pigment content as reported for other seagrasses^47,48^. However, nutrient enrichment at ambient pH caused an increase in the expression of antioxidant (i.e. PrxQ and SOD) and glutathione-related enzymes (i.e. GPX and GR), indicating a response of the plant to stress, possibly due to the proliferation of leaf epiphytes^61^ or as a consequence of internal C-limitation and/or carbohydrate reserve depletion^18,19^, among many other pathways. Although our study cannot provide evidence of the underpinning mechanisms, down- and up-regulation of stress-related genes induced by nutrient addition at low and ambient pH, respectively, strongly suggests that the physiological response of *P. oceanica* to nutrient loading is dependent upon pH levels and vice versa.

Final expression levels of target genes under enhanced nutrient loadings were estimated, separately for each pH level, using the expression of plants collected at time 0 (before adding nutrients) as the reference condition. Since the pre-nutrient addition and the final sampling were performed at different times of the year (May and July, respectively), variation in gene expression could be affected also by seasonality. However, significant variation in target gene expression in plants not exposed to nutrient addition (CTRL) emerged only for GPX at ambient pH and GST at low pH (Fig. 1), suggesting that the response to nutrient addition of most of the genes investigated was not biased by seasonality. In addition, the expression of GPX at ambient pH significantly decreased from time 0 to the final sampling time in the control (Fig. 1), while the opposite pattern emerged under both moderate and high nutrient concentrations, further indicating that variation generated by nutrients was considerably greater than natural variation at the scale of the month.

In summary, our study suggests that nutrient enrichment may have positive effects on *P. oceanica* at low pH, by fostering leaf growth rates. The antagonistic effects of OA and nutrient loading on leaf growth are likely the result of direct effects on the physiology of *P. oceanica* and indirect effects on the epiphytic community. On the one hand, over-expression of genes involved in the transport of nitrogen suggests that nutrient enrichment allows the plant to reduce the nitrogen deficiency caused by OA. On the other, by slightly enhancing epiphyte cover, nutrient inputs at low pH could reduce the exposure of the plant to light stress^52^. Further experimental studies are, however, warranted to identify the mechanisms underpinning the positive effects of nutrients supply on *P. oceanica* at low pH.

OA and nutrients have been previously shown to have synergistic negative effects on other key habitat-forming primary producers in coastal areas. Our results, while supportive of the interactive nature of nutrients and OA, bring some evidence that their net effect on marine primary producers is not necessarily synergistic. This suggests that generalizing the effects of these stressors across species characterized by different life-traits may be a daunting task.

In contrast to our predictions, heavy nutrient loading did not have direct or indirect negative effects on *P. oceanica* close to the CO_2_ vent, since leaf growth and pigment content tended to increase under both moderate and high nutrient levels. In our experiment, nutrient enhancement was likely not effective in generating concentrations high enough to be detrimental to *P. oceanica*. This could also explain the lack of a dose-dependent effect of nutrient enrichment on all the response variables analysed. Identifying the concentration threshold at which the effect of nutrients on *P. oceanica* switch from positive to negative will be a crucial step to gain a comprehensive understanding of the response of this seagrass to OA. In addition, although our study suggests that *P. oceanica* may benefit from enhanced nutrient loading under future pH conditions, other global (e.g. seawater warming) or regional stressors (e.g. sedimentation, inorganic pollution, mechanical disturbance), as well as biotic interactions^16^, may modify the direction and magnitude of their outcome in unpredictable ways. More experimental and modelling (e.g. pathway analysis) work is, therefore, necessary to improve our ability to forecast the response of seagrass beds to future multiple stressor scenarios.

Management of local stressors can represent a valid tool for mitigating the impacts of global changes. For instance, reducing nutrient loading and sedimentation rates has been shown to foster the persistence of canopy-forming seaweeds in the face of OA and seawater warming^3,62^. While this management strategy can be profitable when negative effects of global and local stressors are synergistic, it seems hardly practicable in cases, such as that documented by our study, in which local stressors alleviate negative effects of global stressors^63^. Given the cumulative nature of human impacts and the high small-scale variation in life-traits present in coastal environments, one size fits all strategies are unlikely to be successful for sustaining the functioning of marine ecosystems in the face of climate changes.

## Materials and Methods

### Study site and experimental design

This study was carried out in seagrass meadows at CO_2_ vents off the Castello Aragonese isle (Ischia Island, 40°43′51.01″N, 13°57′48.07″E; Tyrrhenian Sea, Italy), from April 2014 to July 2015. At this site, volcanic vents influence carbonate chemistry, creating a gradient of decreasing pH along a shallow rocky bottom. *P. oceanica* forms dense and continuous meadows along this pH gradient, where shoot density was ~30% higher at low than ambient pH conditions^14^. We identified two levels of pH across the seagrass meadow: ambient pH site and low pH site, the latter reflecting the pH level predicted by the end of the century. In order to measure the relative change in pH between sites, water samples were taken from the water column at 10 dates chosen at random between May and October 2014. At each date, five replicate water samples were taken at each site using a 125 ml bottle, just above *P. oceanica* leaves. Measurements were made using a Mettler Toledo SG2 pH meter, which measures to 0.01 units equipped with an InLab 413 electrode and calibrated regularly using NIST-traceable buffers. Although this approach does not measure the total hydrogen ion concentration, it measures the relative change in pH between sites^64^. The average pH (±SE) at the ambient site was 8.09 ± 0.006 while that at the low pH site was 7.78 ± 0.047 (*n* = 50).

Seawater temperature was continuously monitored using a HOBO data logger, which was positioned between the two sites throughout the experiment. Temperature varied between 14 and 26 °C, with warmest water occurring in August and coldest water temperature in February–March. Temperature is not expected to differ between sites, at a depth of 2.5–3.5 m. The salinity regime around Ischia is typical for Tyrrhenian coastal water and varies generally between 37.0 and 38.5^65^.

The effects of nutrient supply (control vs. moderate vs. high) and OA (ambient vs. low pH) on *P. oceanica* and the associated epiphytic community were evaluated in an orthogonal combination. In April 2014, nine 50 × 50 cm plots, about 3 m apart, were marked at their corners with iron rebars at a depth of ~3 m within *P. oceanica* patches, at each of the two experimental sites. Three plots were then randomly assigned to each of three nutrient addition levels (control vs. moderate vs. high), for a total of 18 replicate plots (2 pH levels × 3 nutrient levels × 3 replicated plots). Elevated nutrient enrichments were designed to replicate conditions comparable to those recorded in urban areas^21^. Nutrient levels were elevated using slow release fertilizer pellets (Osmocote 6 months, 17:11:10 N:P:K) contained in plastic net bags (1-mm mesh size). The high and moderate levels of nutrient addition were, respectively, achieved by deploying 400 g (three mesh bags containing 133 g each) and 200 g (three mesh bags containing 67 g each) of fertilizer, fixed in the middle of each plot by means of iron rebars. Nutrient bags were suspended within the *P. oceanica* canopy, at a distance of ~10 cm from the bottom, and replaced every two months in order to ensure the maintenance of experimental conditions. The weight of fertilizer in each nutrient bag was measured at the third decimal by means of a precision scale before deployment. Upon retrieval, nutrient bags were dried in a muffle for 28 hours at 60 °C and the amount of fertilizer that had not dissolved was re-weighted in order to estimate the average nutrient release rate per day over the duration of the experiment^66^. The amount of fertilizer released (g · m^−2^ · day^−1^ nitrogen and phosphate, based on volumetric ratio) was significantly greater under high (nitrogen: 1.923 ± 0.135; phosphate: 1.244 ± 0.087, n = 24) than under moderate nutrient supply (nitrogen: 1.175 ± 0.095; phosphate: 0.760 ± 0.062, n = 24) (*F*_1,8_ = 101.3151; *P* < 0.001). By contrast, there was no difference in the amount of nutrient released between pH levels (see Supplementary Fig. S2). In addition, in order to estimate the concentration of nutrients, seawater samples were taken from the water column in each experimental plot using a 60 ml syringe, at three dates chosen randomly across the study period (May 2014, June and July 2015). Samples were immediately filtered (0.45 μm) and frozen prior to transport to the laboratory for analysis. At ambient pH, total inorganic nitrogen concentrations (μmol/L nitrates, nitrites and ammonia) were 3.85 ± 0.612, 3.84 ± 0.52 and 5.29 ± 0.63 and phosphate concentration (μmol/L) were 0.103 ± 0.02, 0.174 ± 0.031, 0.172 ± 0.044 for control, moderate and high nutrient treatments, respectively (data are mean ± SE values averaged across the three sampling dates; *n* = 18). At low pH, total inorganic nitrogen concentrations were 4.46 ± 0.91, 5.44 ± 0.91 and 5.94 ± 0.65 and phosphate concentration were 0.172 ± 0.044, 0.290 ± 0.075, 0.474 ± 0.146 for control, moderate and high nutrient treatments, respectively. Natural nutrient concentrations at this site are comparable to those recorded in other peri-urban areas in the NW Mediterranean^21,67^ and are, therefore, unlikely to be limiting for *P. oceanica*. This species forms, in fact, luxuriant meadows in many oligotrophic areas across the Mediterranean^68^.

### RNA extraction and cDNA synthesis

For each of the 18 experimental plots, 3 intermediate leaves (the second-rank leaf in the shoot) were randomly sampled at the beginning (April 2014) and at the end of the experiment (July 2015). Tissue from each leaf (at least a triplicate for each site and for each condition) was collected and rapidly cleaned from epiphytes with a razor blade, towel-dried and immediately stored in RNAlater© tissue collection solution (Ambion, life technologies). Samples were then transported to the laboratory, preserved one night at 4 °C and stored at −20 °C until RNA extraction. For the RNA extraction, portions (about 5 cm) of seagrass leaf tissue were grinded into a fine powder with mortar and pestle in presence of liquid nitrogen. About 100 mg of powered tissue were used for the RNA extraction using AurumTM Total RNA Mini Kit (BIO-RAD) as in Mazzuca, *et al*.^69^. After lysis solution, samples were homogenized using the Qiagen Tissue Lyser and Tungsten Carbide Beads (3mm) (Qiagen) for 3 min at 20.1 Hz. RNA quantity was assured by Nano-Drop (ND-1000 UV-Vis spectrophotometer; NanoDrop Technologies) monitoring the absorbance at 260 nm, while purity was determined by monitoring the 260/280 nm and 260/230 nm ratios using the same instrument. Both ratios were about 2.0. All samples were free from protein and organic solvents used during RNA extraction. RNA quality was evaluated by agarose gel electrophoresis that showed intact RNA, with sharp ribosomal bands. Total RNA (500 ng) was retro-transcribed into cDNA with the iScriptTM cDNA Synthesis Kit (BIO-RAD) following Dattolo, *et al*.^52^, using the GeneAmp PCR System 9700 (Perkin Elmer). The reaction was carried out in 20 µl final volume with 4 µl 5 × iScript reaction mix, 1 µl iScript reverse transcriptase and DNase-free H_2_O. The mix was first incubated 5 min at 25 °C, followed by 30 min at 42 °C and finally heated to 85 °C for 5 min.

### Reverse transcription-quantitative polymerase chain reaction (RT-qPCR)

Expression level analyses were performed for specific genes of interest (GOIs) (See Table 3 and Supplementary Table S1 list selected genes of interest, their functions and primer information): the glutathione related enzymes glutathione synthase (GSH-S), glutathione peroxidase (GPX), glutathione reductase (GR) and glutathione S-transferase (GST), the antioxidant enzymes catalase (CAT), superoxide dismutase (SOD), luminal binding protein (LBP) and Peroxiredoxin Q (PRXQ)^27,70^ and three nitrate transportes (NRT1_6.3, NRT1_2.13 and NRT2). Glutathione-related and the other antioxidant enzymes were selected in order to study the cellular response to oxidative stress, while nitrate transporters were selected to investigate variations in nitrogen uptake upon nutrient-enrichment. Primers for three nitrate transporters were designed with primer 3 (http://bioinfo.ut.ee/primer3/) and validated as in Serra, *et al*.^71^. Oligo for the other genes are reported in Lauritano, *et al*.^27^ (Table 3). RT-qPCR was performed in MicroAmp Optical 384-Well reaction plate (Applied Biosystem, Foster City, CA) in a Viia7 real-time PCR system (Applied Biosystem) as in Lauritano, *et al*.^72^. Plants collected at T0 were used as the control condition. Gene expression levels were analyzed using the REST tool (Relative Expression Software Tool)^73^. Three biological replicates were used for each site and condition, and three technical replicates were used for each biological replicate. Data were normalized using as reference genes the three most stable genes in these conditions^27^. In a previous study a panel of seven putative reference genes (RGs) was in fact screened (eukaryotic initiation factor-4A, ubiquitin, ribosomal protein L23, elongation factor 1-alpha, glyceraldehyde 3-phosphate dehydrogenase, ribosomal RNA 18 S and ubiquitin-conjugating enzyme NTUBC2) and results showed that the most stable were L23, eukaryotic initiation factor-4A, ribosomal protein L23 and ubiquitin-conjugating enzyme NTUBC2^27^. Statistical analyses were performed using GraphPad Prim statistic software, V4.00 (GraphPad Software).

**Table 3:** List of selected genes of interest, their function and primer information.

**Glutathione-related Enzymes**	**Functions**	**Primers**
Glutathione S-transferase (GST)	Detoxification	Lauritano *et al*.^27^
Glutathione peroxidase (GPX)	Glutathione oxidation	Lauritano *et al*.^27^
Glutathione synthase (GSH-S)	Glutathione synthesis	Lauritano *et al*.^27^
Glutathione reductase (GR)	Glutathione reduction	Lauritano *et al*.^27^
**Antioxidant enzymes**	**Functions**	**Primers**
Catalase (CAT)	Detoxification free radicals	Lauritano *et al*.^27^
Superoxide dismutase (SOD)	Detoxification free radicals	Lauritano *et al*.^27^
Peroxiredoxin Q (PrxQ)	Antioxidant	Lauritano *et al*.^27^
Luminal binding protein (LBP)	Antioxidant/Stress protein	Lauritano *et al*.^27^
**Nitrate transporters**	**Functions**	**Primers**
NRT1 6.3	Displays dual affinity for nitrate in the high and low affinity ranges, is involved in nitrate sensing	See supplementary table
NRT1_2.13	Low-affinity nitrate transporters	See supplementary table
NRT2	High-affinity nitrate transporter	See supplementary table

### Chlorophylls and carotenoids determination

At the end of the experiment (July 2015), the content of chlorophylls and carotenoids was assessed in three leaves (the second youngest leaf of different shoots), randomly sampled in each experimental plot. Leaves were washed, scraped with a razor blade to remove epiphytes and freeze-dried. Dried leaves were ground for 15 sec in a steel balls mill (Retsch GmbH & Co. KG, Haan, Germany) cooled with dry ice. Leaf powder was stored at − 30 °C until analysis.

Aliquots of 2 mg powder were extracted with 2 mL of acetone:Tris 0.5 M (80:20, v-v), adjusted to pH 7.8 with HCl, by mixing on a magnetic stirrer for 30 min in a cold room (5 °C). After centrifuging for 5 min at 7750 g, 5 °C, supernatant absorbance was measured spectrophotometrically at 470 nm, 537 nm, 647 nm, and 663 nm, against a solvent blank. Calculations, corrected for anthocyanins, were made after Sims and Gamon^74^. All extractions were repeated four times.

### Plant growth and epiphyte assemblage structure

Leaf growth rate was measured in June 2015, at the seasonal peak of growth. *In situ*, all leaves of three shoots, randomly chosen within each plot, were punched at the same time, just above the ligula of the most external leaf, with a hypodermic needle^75^. Marked shoots were collected after 30 days (July 2015, at the end of the experiment). In the laboratory, epiphytes were gently removed with a razor blade. Leaf tissue of each shoot was divided into newly produced (i.e. leaf material below the hole) and older tissue (i.e. leaf material above the hole) and dried at 60° for 24 h. Leaf growth rate (mg DW · shoot^−1^ day^−1^) was expressed as the weight of new tissue produced divided by the time elapsed between the two sampling events (30 days). Specific growth rate (%; day^−1^) was calculated as the shoot growth rate divided by shoot biomass per 100.

We used the same shoots to estimate epiphyte abundance on leaves. At the end of the experiment (July 2015), prior to measure leaf growth, epiphyte abundance was visually estimated by examining the oldest part (the apical 10 cm from the tip) of the two external leaves of sampled shoots. Animals and macroalgae were identified at the levels of genera or species, when possible, under a dissecting microscope. Abundance was expressed as percentage cover of the proportion of the leaf surface colonised by each species^67,76^. Data from the two leaves within each shoot were then averaged and covers standardized per 10 cm^2^ of leaf area. For analysis, macroalgal epiphytes were divided into calcified (mostly encrusting corallines) and non-calcified forms.

### Statistical analysis

The effects of OA and nutrient treatments on seagrass physiology (photosynthetic pigments) and plant growth were tested using a two-way ANOVA. The model included two factors: pH (fixed, with two levels: ambient and low pH), nutrient addition (fixed, with three levels: control, moderate and high). Cochran’s C-test was used to check for homogeneity of variances and, when necessary, data were log- or square root transformed. All variables were also individually checked for normality, using both an exploratory data analysis procedure (Q-Q plots) and a parametric test (Shapiro Wilks test) and, when necessary, data were transformed to achieve normality (see Supplementary Fig. S3). Student-Newman-Keuls (SNK) tests were used for comparison of the means.

Effects of OA and nutrient treatments on epiphytic assemblages were tested by means of a permutational multivariate analysis of variance (PERMANOVA)^77^ performed on a Bray-Curtis dissimilarity matrix of square root transformed data. The model included three factors: pH (fixed, with two levels: ambient and low pH), nutrient addition (fixed, with three levels: control, moderate and high) and plot (random, nested in pH × nutrient addition). Pairwise *a posteriori* comparison was performed to assess differences among factor levels. Permutational analyses of multivariate dispersion (PERMDISP) were performed to evaluate whether different experimental treatments caused changes in the spatial heterogeneity of epiphytic assemblages. Data were square root transformed to avoid significant multivariate dispersions of data.

Finally, components of the epiphyte community were divided into broad morphological groups of encrusting coralline algae, non-calcified epiphytic algae and total epiphytic assemblage (algae plus animals). The percentage cover of each of the two epiphytic morphological groups (calcified and non-calcified), as well as that of the entire epiphyte assemblage, was analysed using a three-factor ANOVA with the same design described for PERMANOVA.

### Data Availability

The datasets generated during and/or analysed during the current study are available from Figshare https://figshare.com/s/601a42447636b5a2abe7.

## Electronic supplementary material

Supplementary information
