# Supplementary material for: Nutrient Loading Fosters Seagrass Productivity Under Ocean Acidification
Source: Sci Rep. 2017 Oct 23;7:13732. doi: 10.1038/s41598-017-14075-8 (PMC5653774; doi:10.1038/s41598-017-14075-8)
Supplement: Supplementary file 1 — Supplementary information [file 41598_2017_14075_MOESM1_ESM.pdf]

NUTRIENT LOADING FOSTERS SEAGRASS PRODUCTIVITY UNDER OCEAN ACIDIFICATION

Authors

Chiara Ravaglioli[1*], Chiara Lauritano[2], Maria Cristina Buia[2], Elena Balestri[1], Antonella Capocchi[1], Debora Fontanini[1], Giuseppina Pardi[1], Laura Tamburello[1], Gabriele Procaccini[2**], Fabio Bulleri[1**]

Table S1. List of nitrate transporters investigated, their oligo efficiencies, correlation factors and primers.

Nitrate transporters	Oligo Efficiency	Correlation factor	Primers (forward and reverse, 5'-3'')
NRT1_6.3	2	0.992	AATCACCCAGCTCCTCATGC - CAGCCCGGTAGTTCTTGAG
NRT1_2.13	1.81	0.996	GTTGTTCCTAAGCACGCTGG - GTCGTACAGCTTCCCCATGT
NRT2	1.95	0.999	CATGGGTGCATCTTCCCTGT - GCTACCACCCTTCGCTTCTT

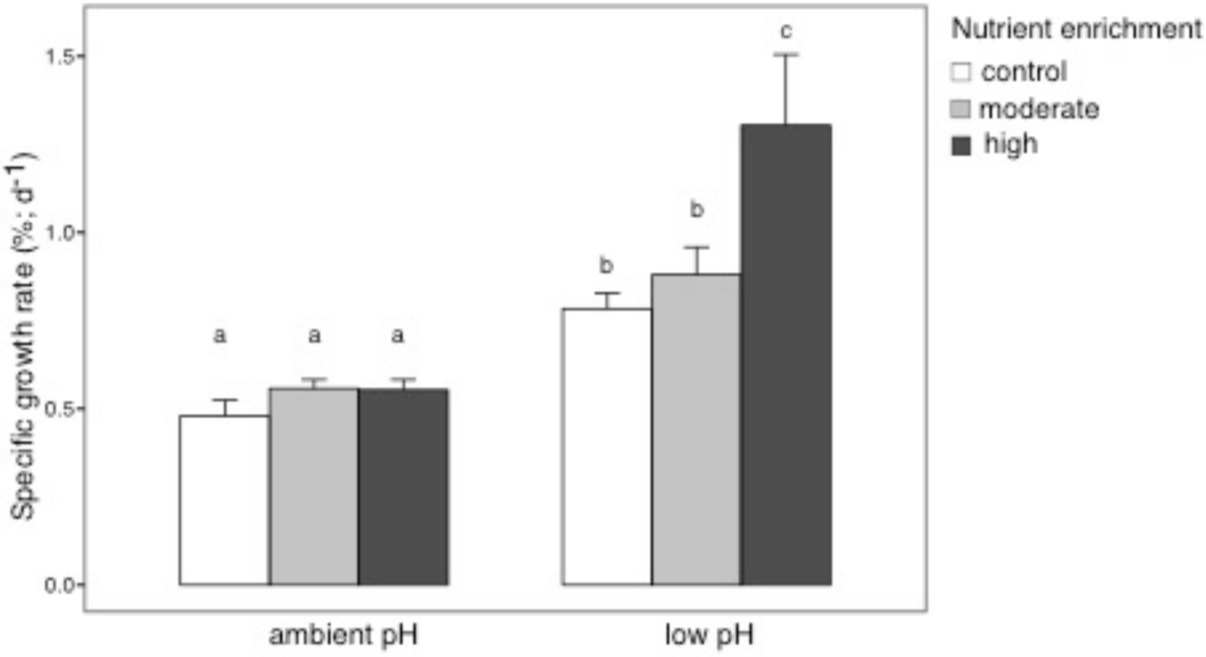

Figure S1 Specific leaf growth of *P. oceanica* (%; day^{-1}, mean +SE, n=9) for different combinations of pH (ambient and low pH) and nutrient enrichment (control, moderate, high). Letters above columns indicate the outcome of SNK tests; different letters indicate significant differences. Letters show the comparison among nutrient treatments within each pH condition.

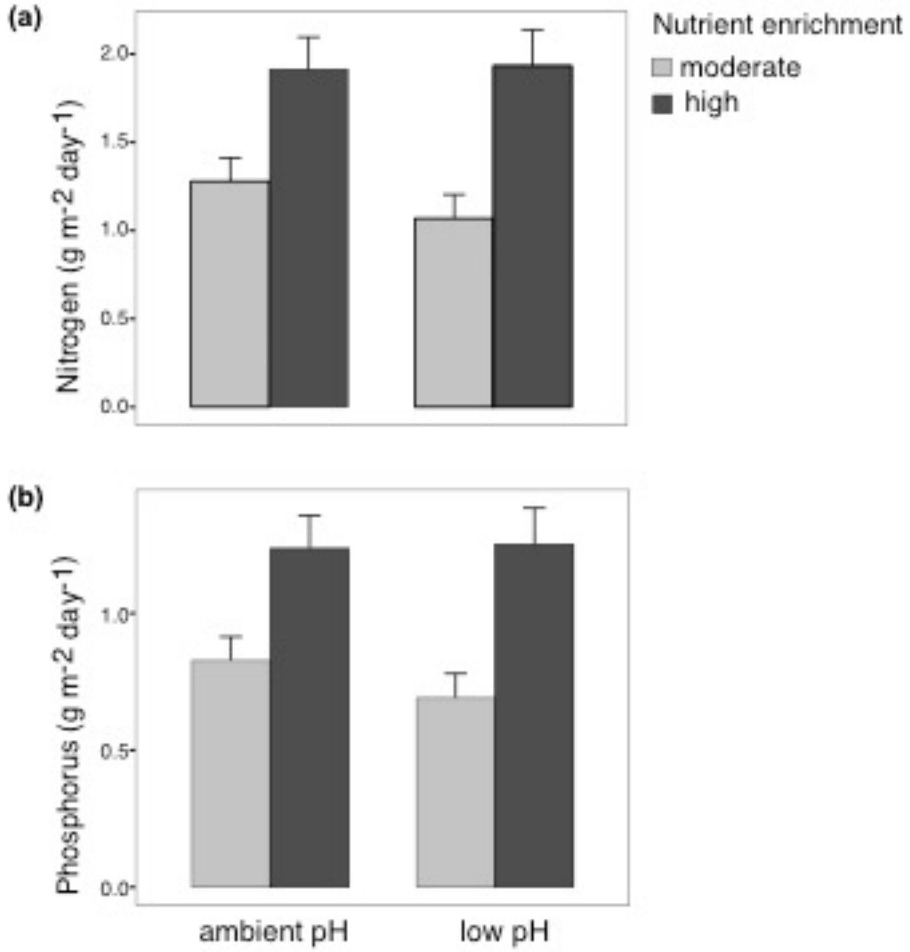

Figure S2 Average daily release of nitrogen (a) and phosphate (b) (g/m^2 * day, mean +SE, n=12) from nutrient bags for different combinations of pH (ambient and low pH) and nutrient enrichment (moderate and high).

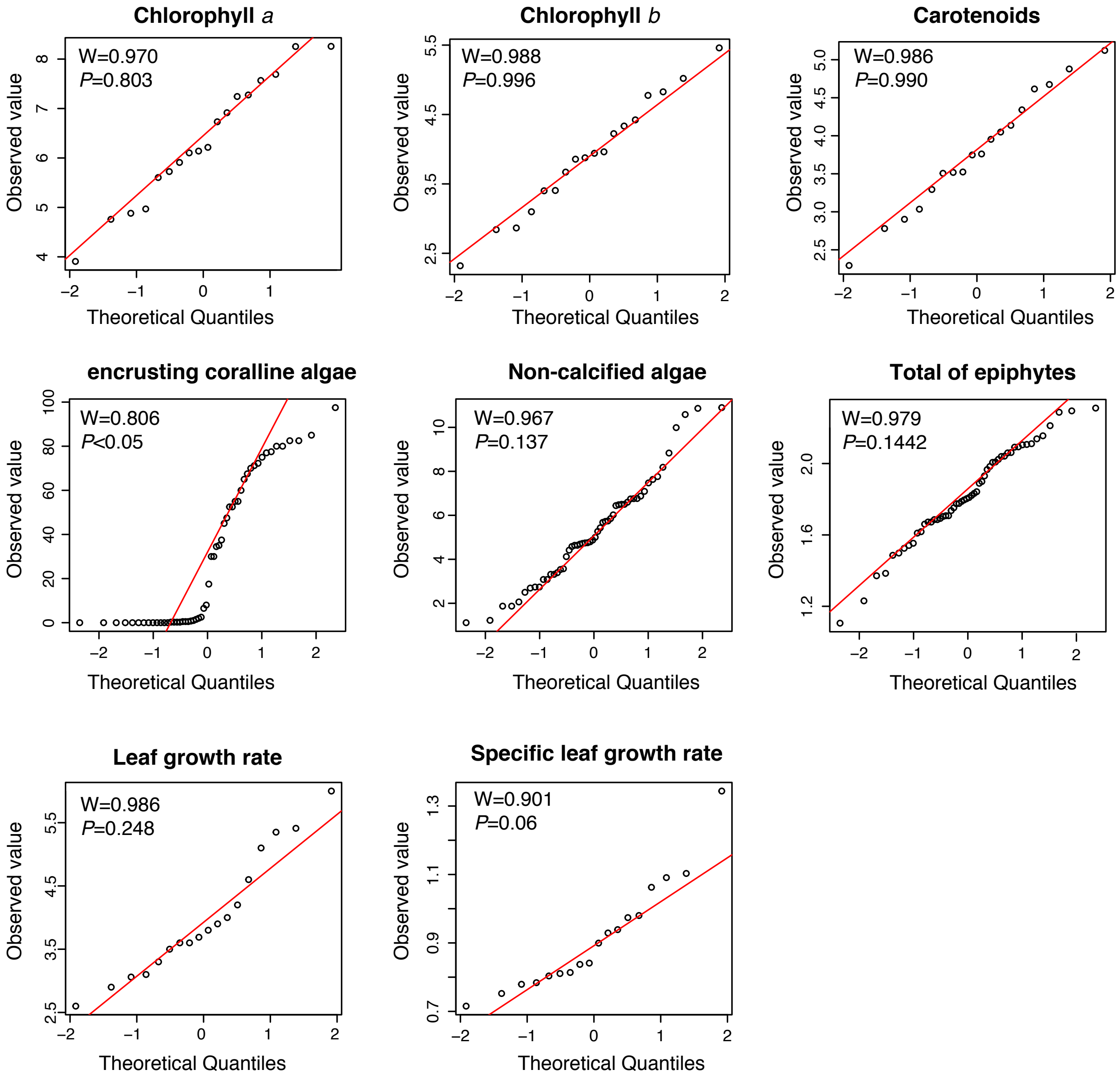

Figure S3 Q-Q plots and Shapiro-Wilks test results (W) for normality of all the response variables analysed
